# Next generation of astronauts or ESA astronaut 2.0 concept and spotlight on immunity

**DOI:** 10.1038/s41526-023-00294-z

**Published:** 2023-06-28

**Authors:** Pauline Jacob, Christian Oertlin, Bjorn Baselet, Lisa S. Westerberg, Jean-Pol Frippiat, Sarah Baatout

**Affiliations:** 1 Stress Immunity Pathogens Laboratory, UR 7300 SIMPA, Faculty of Medicine, Université de Lorraine, Vandœuvre-lès-Nancy, France; 2 Karolinska Institutet, Department of Microbiology Tumor and Cell biology, Stockholm, SE-17177 Sweden; 3 Radiobiology Unit, Belgian Nuclear Research Centre, SCK CEN, Mol, Belgium; 4 Department of Molecular Biotechnology, Gent University, Gent, Belgium

**Keywords:** Experimental models of disease, Immunological disorders, Immunological disorders

## Abstract

Although we have sent humans into space for more than 50 years, crucial questions regarding immune response in space conditions remain unanswered. There are many complex interactions between the immune system and other physiological systems in the human body. This makes it difficult to study the combined long-term effects of space stressors such as radiation and microgravity. In particular, exposure to microgravity and cosmic radiation may produce changes in the performance of the immune system at the cellular and molecular levels and in the major physiological systems of the body. Consequently, abnormal immune responses induced in the space environment may have serious health consequences, especially in future long-term space missions. In particular, radiation-induced immune effects pose significant health challenges for long-duration space exploration missions with potential risks to reduce the organism’s ability to respond to injuries, infections, and vaccines, and predispose astronauts to the onset of chronic diseases (e.g., immunosuppression, cardiovascular and metabolic diseases, gut dysbiosis). Other deleterious effects encountered by radiation may include cancer and premature aging, induced by dysregulated redox and metabolic processes, microbiota, immune cell function, endotoxin, and pro-inflammatory signal production^1,2^. In this review, we summarize and highlight the current understanding of the effects of microgravity and radiation on the immune system and discuss knowledge gaps that future studies should address.

## Introduction

The European Space Agency (ESA) aims to expand the reach of humanity into space. Space is an extremely hostile environment that poses threats to human health. One such threat is the well-established induction of immune system alterations in astronauts during spaceflight that persist after return^3–6^. The immune system protects against invading pathogens, toxins, and cancer cells and dysregulation thereof can lead to drastic consequences during space missions. Therefore, it is warranted to put a spotlight on current research that encompasses the immune system, space, and space analogs to assess risks, formulate countermeasures, and direct future research.

In 2021, ESA launched the Terrae Novae program to lead Europe’s human journey into the Solar system using both robots as precursors as well as astronauts, and to return the benefits of exploration back to society. Terrae Novae has the literal meaning of ‘New Worlds’ that includes the three ESA exploration destinations: Low Earth Orbit (LEO), Moon, and Mars. It is therefore expected that a new generation of astronauts will be associated with the Terrae Novae program ensuring a broad demographic representation and supporting a wide range of mission profiles. These astronauts will undergo rigorous pre-flight screening to predict and mitigate their reactions to the space environment, and preserve their well-being using inflight tools available to them. These tools will range from managing group dynamics to improved communication facilities, interfacing with diagnostic devices, and artificial intelligence to assist with inflight health monitoring.

In this context, ESA invited the scientific community to prepare white papers, of which one was focused on the immune system, to identify key knowledge gaps, and to provide recommendations for future research. The white paper for the immune system was thereafter reviewed by the space scientific community in Immunology and amended accordingly (https://esamultimedia.esa.int/docs/HRE/12_HumanResearch_HumanPhysiology.pdf). In this review, we present an overview of current knowledge about the effects of spaceflight on immune cells and the immune system in general, as well as the main outcomes of this white paper.

## Effects of space conditions on the immune system

### General overview of the immune system

Skin and epithelias are the largest organs of the human body and represent a first line of defense^7^. Non-immune cells present in these tissues are equipped with pattern recognition receptors (PRR) of which the activation leads to the production of chemokines and cytokines that activate the immune system^8^. Furthermore, the skin contains resident myeloid and lymphoid immune cells including T lymphocytes, monocyte-derived dendritic cells as well as macrophages^8^. While the barrier function of these tissues provides protection against infiltration of pathogens, the gut provides an environment that hosts a large microbiome^9^. This microbiome plays a role in central nervous system regulation, energy metabolism but also in the immune system^9^. The immune system interacts with numerous systems (e.g., the central nervous, the cardiovascular, the musculosketal, the reproductive, the respiratory, and the digestive systems) of which the interplay with gut microbiome is of particular importance as its dysregulation can lead to disease such as inflammatory bowel disease (IBD)^9^.

**Fig. 1:**
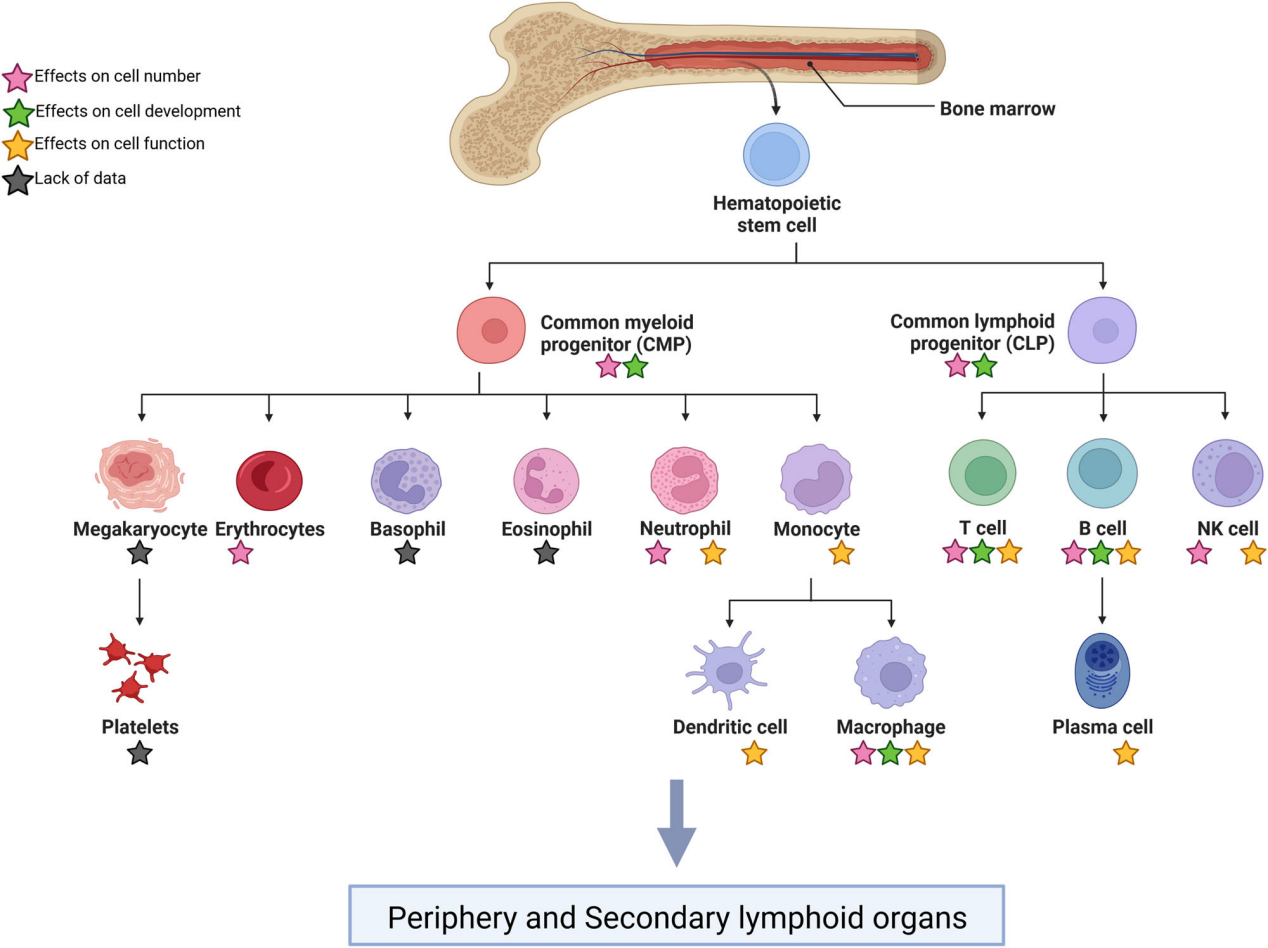
Effect of spaceflight-associated stressors on immune cell number, development, and function. Immune cells derive from self-renewing hematopoietic stem cells. These cells differentiate into common myeloid progenitor cells (CMP) and common lymphoid progenitor cells (CLP). CMP form megakaryocytes, erythrocytes, basophils, eosinophils, neutrophils, and monocytes which can give rise to dendritic cells and macrophages, whereas CLP differentiate into T cells, B cells which can give rise to plasma cells, and natural killer (NK) cells. Stars indicate if spaceflight-associated stressors affect the number (pink), the development (green), and/or the function (yellow) of each cell type. A black star indicates a lack of data. Created with BioRender.com.

Immune cells contribute to innate and adaptive immunity. Granulocytes, monocytes, macrophages, and NK cells are part of the fast-responding innate immune system. Dendritic cells bridge the innate immune response to the slow-responding and highly specific adaptive immune system consisting of B and T cells. All these cells derive from self-renewing hematopoietic stem cells (HSC). HSC differentiate into common myeloid progenitor cells (CMP) as well as common lymphoid progenitor cells (CLP). CMP form megakaryocytes, erythrocytes, basophils, eosinophils, neutrophils, monocytes, dendritic cells, and macrophages whereas CLP differentiate into T cells, B cells, and natural killer (NK) cells (DC) (Fig. 1).

### Effects on immune cell differentiation

A number of studies have analyzed the impact of spaceflight on the development of immune cells^6^. A decrease in the number of granulocyte and monocyte progenitors was reported in rodents that have been in space or subjected to anti-orthostatic suspension (AOS, a model used to mimic physiological changes observed during a spaceflight)^10–12^. The inflight culture of human CD34^+^ progenitors revealed an inhibitory effect on red blood cell production^13^. A study using an amphibian model^14^ showed that the transcription of IgM heavy chain was increased, which was associated with the decreased expression of the lymphoid-determining transcription factor Ikaros^15^, thereby suggesting an effect on B cell development. This perturbation of B lymphopoiesis was confirmed in mice submitted to 21 days of AOS. In this study, a reduced proportion of CLPs, pro-B cells, and pre-B cells along with an alteration of femoral bone structure was noted^16^. In the same way, analysis of femurs of mice flown for one month onboard the BION-M1 satellite showed an altered expression of numerous proteins required for bone and immune cell development^17^. Furthermore, a reduction in B cells in the bone marrow and spleen one week after landing was evidenced^17^. T lymphopoiesis is also altered by microgravity, as a decrease in double-positive (CD4^+^CD8^+^) and single-positive (CD4^+^ or CD8^+^) T cell maturation stages were observed when murine fetal thymuses were cultivated under simulated microgravity (sµG)^18^. A significant suppression of thymopoiesis was also noted in 16 astronauts upon return from spaceflight^19^. Analysis of the impact of being conceived and born under increased g force (2 G) on the murine T cell antigen receptor (TCR) repertoire revealed a disruption in TCR signaling and in the diversity of this receptor binding sites^20^. Finally, it was shown that a model mimicking socioenvironmental stress experienced by astronauts also modifies the murine TCR repertoire created during T lymphopoiesis^21^.

### Effects on mature immune cells

#### Neutrophils

Neutrophils are the most abundant type of granulocytes and the first responders at the site of an infection. Neutrophils are recruited in large numbers from the blood, through the endothelium, to the infected tissue where they release vesicles loaded with proteolytic enzymes and antimicrobial peptides. Upon encountering bacteria, neutrophils phagocytose and kill them by production of reactive oxygen species. Abnormalities in any aspects of neutrophil development and/or function induce immunodeficiency or aberrant inflammatory reactions^22^. It has been shown that neutrophil number increases after short and long spaceflights^5^ but neutrophils had less capacity to phagocyte pathogens^23^. A recent study showed that neutrophil to lymphocyte ratio increased when leukocytes from healthy human donors were exposed to sµG^24^. This increase was also noted in 23 astronauts at the end of 6-month ISS expeditions^24^ and in 20 healthy volunteers immediately post-bed rest exposure^25^, indicating an elevated level of inflammation.

#### Monocytes

Monocytes are present in blood and when entering tissues, differentiate into macrophages and myeloid dendritic cells (DCs). The monocyte number is relatively stable during a short spaceflight^26^, but could be increased during longer missions^27^. Thus, the effects on monocyte number seem dependent on mission’s duration. Their function is affected as illustrated by reduced expression of CD62L and HLA-DR involved in migration and antigen presentation. Upon stimulation with lipopolysaccharides (LPS), monocytes from astronauts showed lower secretion of pro-inflammatory cytokines during the flight compared to before launch, thereby confirming the impairment of monocyte function^26^. Another study used the myelomonocytic human cell line U937 as a model for monocytes and showed that the expression of genes involved in monocytes and macrophage function (e.g., TAP2 and ABCC4) are impaired in sµG^28^. Even if this cell line is a myelomonocytic cell line, it is mainly used to study the differentiation and function of macrophages that derive from monocytes thereby explaining why less data are available for monocytes compared to other cell types.

#### Macrophages

Macrophages phagocytose pathogens and present antigenic peptides to activate T cells. There are two main types of macrophages: the M1 type known as pro-inflammatory or classically activated macrophages, and the M2 type known as anti-inflammatory or alternatively activated macrophages. These two types have important roles in immunity but also in tissue homeostasis. Shi et al.^29^ recently showed that sµG and real microgravity (µG) severely reduce the differentiation of macrophages from hematopoietic progenitors. These authors also showed that the polarization into M1 or M2 type are reduced because the RAS/ERK/NFκB pathway is downregulated under sµG and µG conditions. These pathways are important for the differentiation of macrophages but also for their proliferation and function. A reduction of the proliferation of differentiated U937 cells under microgravity was also observed and could be explained by a downregulation of CDC25, a key factor for G2/M transition during cell cycle progression^30^. Another study conducted on a murine macrophage cell line showed a disruption of the cytoskeleton which is crucial for macrophage function^31^. In both M1 and M2 macrophages, an upregulation of beta-actin gene was shown. Additionally, these authors showed a reduction in secretion of the pro-inflammatory cytokine TNFα by M1 macrophages in sµG. On the contrary, the production of reactive oxygen species by macrophages, used to degrade pathogens, was increased under spaceflight conditions, an observation that agrees with the higher inflammatory status noted above^32^. Tauber et al.^33^ showed that a human M1 cell line present a decreased expression of CD14, important for the recognition of pathogens, as well as of ICAM-1, important for cellular adhesion and therefore cell function, under real space conditions. A recent comparison of genetically identical twins of which one spent 1 year at the ISS and the other remained at Earth^34^ indicated elevated pro-inflammatory cytokines TNF-α, IL-1β, and IL-1α in-flight^35^. Post-landing single-cell analysis indicated a progressive decrease in the M1 progenitor monocyte population and a progressive increase in M2 progenitor monocytes when compared to pre-flight data. This suggests that ISS exposure favors pro-inflammatory M1 type macrophage responses leading to prolonged inflammation. Taken together, these findings indicate that microgravity alters macrophage development and functions and may affect M1/M2 polarization.

#### Natural killer cells

Natural killer (NK) cells recognize and kill cancer and virus-infected cells via an immunological synapse and the release of vesicles containing cytotoxic proteins such as granzymes and perforin. NK cells secrete IFNγ and TNFα that can activate the adaptive immune system. A reduction in the cytotoxic capacity of NK cells upon sµG and µG has been reported^36^. While Mylabathula et al.^37^ observed decreased cytotoxicity after 12 h of sµG, Li et al.^38^ reported that effects could be seen only after 48 h. Co-culture of blood-derived NK cells from ISS crew members with K562 leukemia target cells for 24 h showed no decrease in NK cell cytotoxic capacity^39^. Long-term exposure of NK cells to µG was assessed in blood samples from ISS crew members compared to ground control samples. Overall, a decrease in the cytotoxic capacity of NK cells towards target cell lines was reported. Interestingly, astronauts with longer exposure to µG showed a later onset of the reduction in cytotoxic activity indicating a possible adaptation to µG conditions^36^. Degranulation capacities of NK cells were investigated by assessing perforin and granzyme B levels. A reduction of perforin was observed in sµG^36^ but not in blood samples from ISS crew members^36,39^. Granzyme B production seemed not to be affected by µG. To assess whether the activating or inhibitory capacities of NK cells were altered by µG, cell surface receptors were measured. A reduction of both mRNA levels and cell surface expression of the activating receptor NKG2D and inhibitory receptor NKG2A^36^ was observed in NK cells cultivated >24 h in sµG, whereas <24 h culture showed similar expression to control NK cells. Furthermore, cell surface receptors of blood NK cells from astronauts were not affected by µG conditions^36,39^. Lastly, IFNγ was reduced upon 12h^39^ or >48h^36^ sµG culture, whereas TFNα only showed a reduction after 12h^36^. Possible countermeasures against reduced NK cell cytotoxicity upon sµG were explored by Li et al.^38^. The addition of IL-15 but not IL-2 or IL-12 could rescue NK cell cytotoxicity towards K562 leukemia target cells. Moreover, these authors reported that NK cells in 1 G environment post sµG exposure regained cytotoxic capacity. This was also detected in isolated NK cells from ISS crew member blood where cytotoxic capacities were regained after return to Earth^36^. Viral reactivation may be a threat during long-time space missions as varicella-zoster virus DNA was detected during and after spaceflight^40–42^. Some cases resulted in clinical disease manifestation of herpes zoster^41^. Furthermore, an exploratory RNA-seq analysis revealed vulnerability to viral infections during a 12-month period of isolation and confinement at the Concordia station^43^. More research on NK cells is needed as current studies show somehow contradictory results on cell surface marker expression, granulation, and cytokine secretion. Cytotoxic capacities of NK cells towards target cells are affected in both sµG and µG, yet the mechanisms behind this remain to be explored.

#### Dendritic cells

DCs link innate sensing to adaptive immune responses. After sampling antigens in peripheral tissues, DCs mature and migrate to lymph nodes, where they initiate adaptive immune responses by presenting processed antigens in the context of Major Histocompatibility Complex (MHC) molecules to T cells. DCs presenting antigenic peptides on MHC-I molecules activate CD8^+^ cytotoxic T cells whereas antigen presentation on MHC-II molecules activate CD4^+^ helper T cells^44^. Activation of DCs leads to increased cell surface expression of co-stimulatory molecules CD80 and CD86 both of which bind to the co-stimulatory receptor CD28 on T cells leading to T cell activation^44^. The importance of antigen presentation in immune responses place DC function in sµG under scrutiny. The proliferation of DCs from isolated CD34^+^ and CD14^+^ progenitor cells from human blood is reduced under sµG^45^. This was associated with reduced expression of MHC-II molecules and CD80. These findings were also detected when murine JAWS II DCs were cultured in sµG for 14 days, but not after 7 days of culture^46^. DCs isolated from murine spleen and bone marrow showed increased cell number after 16 h in sµG^47^. These results suggest a time-dependent effect of µG on DCs. In blood isolated from an astronaut from the Eneide space mission, DCs generated using isolated monocyte precursors showed reduced MHC-II, CD80, and CD86 cell surface expression which was mirrored by gene expression of these markers. Moreover, gene expression of NF-κB, a transcription factor important for DC activation, was downregulated. These data indicate that DC maturation is reduced after spaceflight while DCs regained normality one-year post spaceflight^48^. sµM do also impact maturation and cell surface marker expression of DCs^46,48^.

#### T cells

T cells are a major component of adaptive immunity and their roles include direct killing of infected host cells, regulation of immune responses *via* cytokine production, and activation of other immune cells. The earliest and essential step of T cell activation is the formation of an immune synapse requiring fine-tuned rearrangements of the cell cytoskeleton^49^. The immune synapse signals towards the nucleus to alter gene expression needed for T cell activation or differentiation into T cell subsets (i.e., T helper cells, T effector cells or T memory cells). In a series of important early studies, the cytoskeletal structures in cultured human cells were found altered in microgravity^50–54^. The actin cytoskeleton state and cytoskeleton-associated proteins was proposed as one of several possible cellular gravisensing systems^49,55^. A drawback of the earlier studies is that many cell biology experiments lacked adequate controls such as 1 G centrifuged control samples and proper experimental conditions (e.g., well-controlled temperature). More recently, alteration of the actin cytoskeleton of human cancer cells exposed to microgravity was demonstrated by life-cell imaging with a compact fluorescence microscope (FLUMIAS) during a parabolic flight^56,57^. The structures or mechanisms that act as ‘gravity responders’ in T cells are still unknown, however, inhibition of lymphocyte proliferation in microgravity is due to alterations occurring within the first hours of exposure to microgravity^58^. Microarray expression analysis revealed altered cytoskeletal gene expression^59^ and overall altered patterns of global gene expression in space-flown human T cells when compared to T cells activated in 1 G during spaceflight^60^. Particularly, impaired induction of early genes, regulated by transcription factors NF-κB, CREB, ELK, AP-1, and STAT as well as HIF-1α and hypoxia-inducible transcripts contributed to T cell inhibition in modeled low gravity^57,60,61^. Analysis of gene expression in T cells from four human donors on board the ISS identified 99 downregulated genes in microgravity^60^. In the context of cytoskeletal regulation, it was especially interesting that a majority of the genes downregulated in T cells exposed to microgravity contained DNA serum response elements (SRE) in the promoters that binds serum response factor (SRF), a direct regulator of cytoskeletal genes^60^. In the NASA twin study, T cells showed the highest number of differently expressed genes, even after the return to Earth^34^. The lymphocyte response towards mitogens in astronauts and cosmonauts is reduced after spaceflight. Human T cells stimulated with the mitogen concanavalin A (ConA) reveal considerably reduced in vitro activation and cell proliferation in microgravity^49^, on a sounding rocket flight^62,63^, and in a rotary bioreactor system^64^. The study of microgravity effects on cultured human T cells on a sounding rocket flight showed that binding of ConA to T cells was not influenced by microgravity, whereas patching of the ConA receptors, a process that involves the actin cytoskeleton to reorganize highly ordered membrane structures, was significantly lower^52^. T cells, pre-exposed to microgravity and stimulated with ConA, show decreased expression of activation receptors including CD25, CD69, and CD71, and reduced inflammatory cytokine secretion and cell proliferation when compared to control T cells exposed to 1 G gravity^64^. Moreover, human lymphocytes undergo apoptosis upon exposure to modeled low gravity^65^. Together, T cells in sµG show marked changes in gene expression and cytoskeletal rearrangement, and further studies are needed to understand the long-term effect of real and sµG on T cell differentiation and T cell responses.

#### B cells

B cells are responsible of humoral immunity. B cells express highly specific and highly diversified receptors, known as immunoglobulins (Ig), or antibodies, that directly bind and neutralize pathogens and toxins. The breath of the Ig repertoire sets the humoral response and therefore, any alteration in the Ig repertoire may lead to immune dysregulation. No change in the number of human B cells was reported during a 6 months stay on the ISS^66,67^ or after 2 months of bed rest^68^. The analysis of the Ig repertoire of mice flown aboard the ISS for a short period of time (21–22 days) indicated that this repertoire was similar between ground control and flight animals^69^. However, Bucheim et al. reported that the IgM repertoire of two out of five healthy cosmonauts who stayed 6 months on the ISS was affected^67^. These modifications of the IgM repertoire persisted up to 30 days after landing. Only marginal changes were observed in the levels of soluble biomarkers of B cell homeostasis, i.e., IgG and IgM levels after 6 months spaceflights measured in the blood of 23 astronauts^66^. To address the B cell response upon infection, researchers have immunized an amphibian animal model^14^, *Pleurodeles waltl*, during a long-term spaceflight (5 months). Analyses of these animals highlighted that the antibody production as well as the somatic hypermutation rate, which diversify antibody binding sites to increase its affinity for the antigen, is affected^70–72^. Recent studies of immunized AOS mice also showed changes in the usage of antibody gene segments^73^. All of these findings indicate that spaceflight can alter the humoral immune response and modify the antibody repertoire but further researches are required to precise the impact of spaceflight on humoral immunity.

## Identified key knowledge gaps

The white paper for the immune system identified six major knowledge gaps.

### Integration of immune system data

Determination of how the different immune cells respond and adapt to long-term exposure to microgravity, ionizing radiation, and psychological stress (>1 year) in a significant number of subjects is of vast importance. Understanding immune suppression (low response to infections) versus hyperinflammation, allergy, cancer, and autoimmunity (often also associated with low response to infections) will be important to develop common and tailored, and precise countermeasures. The latter is important since there may be large variations between individuals in their immune responses^74^. Moreover, this research will increase our understanding of the adaption of the immune system to Space and Earth conditions. These aspects have a wider societal interest, as they could lead to a better understanding of the role of these extreme stress factors in the pathogenesis and pathophysiology of various diseases in our societies which will have to face important climate changes in the near future.

### Contamination, dust, infection, vaccination, autoimmunity

Dust within space vehicles could provoke skin, eye, and other irritations for the astronauts. These are closely connected to allergy and infection, and should be further addressed for long-term missions. Infection and irritation through contamination from microbial or Lunar/Martian material and from microbes in the space habitat is also of relevance. A better understanding of microbiome changes on the immune system should be further evaluated. Indeed, some bacteria can be more resistant to antibiotics^75^ and can increase their virulence when exposed to space conditions^76,77^. Antibiotics may be used during an infection on board a space vehicle. However, an increased antibiotics resistance of bacteria combined with a decrease of antibiotics efficiency, through a change in their pharmacodynamics in space can be experienced^78^. Moreover, microgravity has been shown to promote biofilm formation^76,78,79^ through which bacteria grow and stick to eachother. Biofilms are known not only to increase antibacterial resistance among bacteria but also their ability to infect. The dissemination of antibiotic resistance gene-carrying plasmids among bacteria in gut microbiota is another event that could be affected by stress^79^. Vaccination of astronauts against potential pathogens before and possibly revaccination during spaceflights should be considered to maximize the prevention of infectious diseases^67^ (Fig. 1). There is currently no clear evidence that spaceflight may be associated with a risk of developing autoimmune diseases. However, this possibility should not be neglected because several observations suggest that this type of problem could potentially occur. Indeed, murine medullary thymic epithelial cells (mTECs) were shown to be reduced after 14 days of hindlimb unloading^80^. mTECs express tissue-specific antigens and are critical for removing self-reactive T cells^81^. A model mimicking socioenvironmental stressors encountered during space missions suggested an increase in the self-reactivity of the murine TCRβ repertoire^21^ A decrease in human T helper 1 cell cytokine expression was noted postflight^82^ suggesting a potential T helper 2 cytokine shift that represents a risk for T helper 2 related autoimmune diseases such as rheumatoid arthritis and systemic lupus erythematosus. In the same way, an increase in human platelet to lymphocyte ratio was observed after two months of head-down tilt bed rest^25^. Such increase was shown to correlate with disease activity of systemic lupus erythematosus^83^. Finally, changes in the antibody repertoire of two out of five analyzed cosmonauts were observed during flight and up to 30 days after landing^67^.

### Cancer, radiation, virus reactivation

Cancer is caused by an accumulation of genetic mutations and can be acquired through chronic exposure to environmental stressors as encountered during long-term space missions. Cosmic radiation has been identified as a main threat for cancer development during long-term space missions^2^. Cancer may also develop when the immune system is compromised affecting both anti-tumor responses mediated by NK and T cells and controlled proliferation of immune cells, leading to leukemia and lymphoma. Identifying mechanisms of cancer development and progression during spaceflights is crucial. Since the impairment of immune system activity is crucial for cancer progression, and a healthy immune system can counteract tumor growth, it is considered as very important to elucidate how microgravity may affect the existing balance between immunity and immune escape mechanisms in cancer. Moreover, the role played by cancer microenvironment, namely the stromal components and the extracellular matrix, needs further investigation. Finally, reactivation of latent viruses such as Esptein Barr virus and cytomegalovirus is a threat, especially in a setting of compromised immune cells, and therefore should be carefully monitored during long-term space missions. Metha *et al* analyzed saliva and urine samples for Epstein-Barr virus, varicella-zoster virus, herpes simplex virus type 1, and cytomegalovirus and identified virus sheeding in 8 of 23 ISS crew members^84^. This data emphasize the importance of careful monitoring of crew members for immune system alterations, and reactivation of latent viruses will serve as a critical readout for intervention using countermeasures.

### Individual factors, precision medicine, and countermeasures

**Fig. 2:**
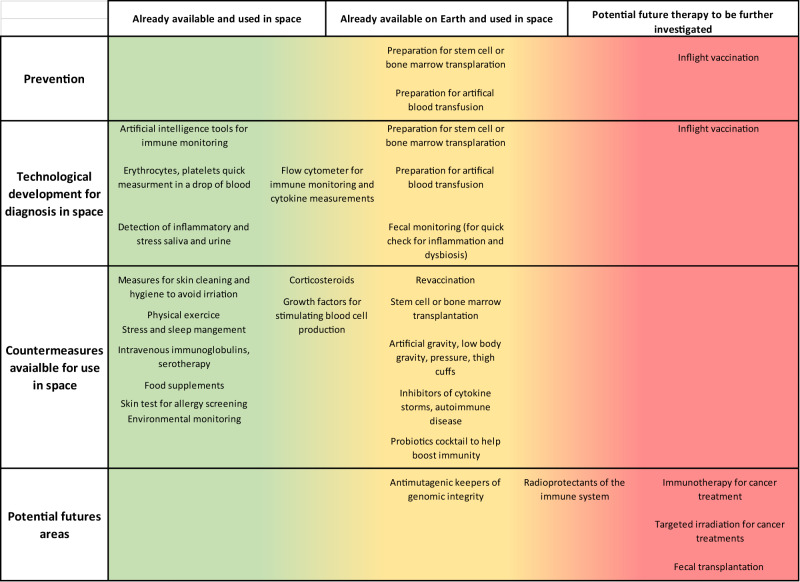
Current and future immune-related countermeasures in space. Prevention, technological development for inflight diagnosis, countermeasures available for use in space, and potential future countermeasures. The green background indicates those already available and used in space, the yellow background those that are not yet used in space, and the red background points out potential future therapy to be further investigated.

Investigating the molecular mechanisms behind why and how individuals respond differently genetically as well as epigenetically to space stressors has been pointed out as of high importance. This issue is relevant to develop a better understanding of human adaptation to spaceflight and could also potentially be of interest to consider in the future selection of astronauts and crew composition for long space exploration missions. Indeed, personal response, stress management, and operational improvements will have a direct impact on the general immune response of the astronauts as highlighted in a recent review^85,86^. While it is challenged by ethical issues, genetic predisposition seems to be a topic of increasing interest. Precision medicine is highly applicable for individual treatment strategies and countermeasures on Earth and could be beneficial to be tailored to each astronaut. The development of predictive biomarkers of immune sensitivity should be identified, implemented, and regularly monitored during long-term space missions. These potential biomarkers could for example include quantification of cytokines and neutrophil/lymphocyte ratio to monitor inflammation status^24^, and viral reactivation as a biomarker of spaceflight-induced alteration of cell-mediated immunity^40,87,88^ (Fig. 2).

### Omics approaches to study the immune system

Recent integration of currently available Omics data from astronauts at ISS with low number of individuals and larger number of data sets from animal and cell-based models in space and at ground-based facilities have revealed similarities and differences when comparing human versus model systems and space versus Earth^34,89^. This multiomics approach identified significant enrichment for mitochondrial processes, innate immunity, and chronic inflammation^89^. Including comparison to the NASA twin study, mitochondrial stress is a consistent phenotype of spaceflight and highly relevant for immune cells. It will also be important to increase knowledge of alterations in RNA transcription and translation (transcriptomics) as well as epigenetic patterns for gene regulation in the various types of immune cells, immune cell repertoire analysis, proteins (proteomics), and metabolites (immune cell metabolomics). Finally, it will be necessary to look for correlations with Omics data deduced from other systems, as the immune system interacts with and may be regulated by many other systems.

### Technology development

Monitoring of the immune system during short- and long-term space missions has been identified as a key knowledge gap and is tightly linked to the development of new technologies (Fig. 2). Space analog researches and ground-based facilities have proven to be valuable for the prediction of specific aspects of the immune system that will be useful to monitor. These techniques include flow cytometry, sample preparation and storage for Omics analysis, sampling approaches (blood, saliva, urine, feces), microscopy (low and high resolution), artificial intelligence/machine learning, and realtime PCR analysis for gene expression^90^. In this context, it is to note, for example, that a recent study showed that genotoxic stress in humans can be monitored by quantifying the amount of DNA double-strand breaks in lymphocytes from a simple finger prick upon exposure to galactic cosmic ray components^91^.

## Identified priorities for the space program: recommendations in short, middle, and long-term

Four main recommendations emerged from the consulted space scientific community in Immunology to help addressing identified key knowledge gaps.

### Recommendation 1: standardization of methods for immunity-related experiment design, performance, and analyses

To better understand immune changes, there is an emerging need to combine analysis of immune cells from ground-based and space platforms, and also across species where possible. Ground-based platforms have the advantage of isolating one parameter to study, for example altered gravity or ionizing radiation, and to perform quantitative analysis of a large number of samples and conditions. Space-based platforms have the advantage of exposing samples to real space conditions, but is often limited to qualitative analysis of a limited amount of samples. Therefore, there is a need to continue to use and develop ground-based and space platforms for the analysis of immune cells and immune system adaptation/alteration. Standardization of culture conditions (cytokine, growth factor, antibody stimulation), flow cytometry-based parameters, and bioinformatics analysis for Omics data is urgently needed to allow efficient comparisons between studies thereby facilitating the discovery of mechanisms underlying observed changes. It is also recommended that the raw data files for analysis are made publically available to the scientific community in a comparable and usable form.

### Recommendation 2: set up a database of results from ground and space-based integrative human research

A data management and distribution system should be established in coordination with major European stakeholders, ESA and EC, to make these data freely accessible to the scientific community. Protocols should be established for disclosure of anonymous crew health data, retrospectively and prospectively, to qualified researchers. Maximum exploitation of currently available resources on Earth (ESA ground-based facilities) and in space, as well as respective databases is mandatory. Examination of these data will provide the basis for a critical, high-quality health care for crews in orbit and will yield solutions for medical challenges for long-term spaceflights. This shared database would provide the basis for well-conceived and evidence-based decisions to physiological concerns as well as for the development of a new generation of integrated countermeasures. NASA GeneLab is the first comprehensive space Omics database that aims to optimize scientific return from spaceflight and ground simulation experiments funded by multiple space agencies around the world^92^. ISSOP (International Standards for SpaceOmics Processing) has been founded as an international consortium of scientists to enhance guidelines for Omics analysis between space biologists globally including European researchers^93^. While ESA does not yet have its own sample-sharing schemes, it encourages multinational spaceflight experiments with sample-sharing between European researchers and around the world.

### Recommendation 3: develop new tools to monitor the immune system

Monitoring of the immune system and immune cells has been identified as a key knowledge gap and is tightly linked to the development of new technologies for detailed analysis and monitoring of astronauts during long-term space missions. Space analog research and ground-based facilities have proven to be valuable for the prediction of specific aspects of the immune system that will be useful to monitor and therefore should be further implemented in this effort. There is currently minimal clinical laboratory equipment at ISS and therefore, devices that measure white blood cells will be useful for monitoring the immune health of the astronauts. A commercial blood analyzer (‘Hemocue’ WBC/diff, Sweden) for fingerstick blood sample has been tested and initial analysis suggests that it functions well in microgravity and at ISS^94^. Quantifying only cells in blood samples may not suffice; more sophisticated analyses will likely be needed. These methods and techniques should be developed to monitor the differentiation and developmental stages of immune cells, and also include analysis of the expanding number of subtypes within one immune cell population. For B and T cells, analysis of the Ig and T cell receptor (TCR) repertoire may provide much more in depth information compared to only the number of cells. Individual measurements over time versus common variations among astronauts, cells, and animal models would be important. It will be important to identify immune “signaling hubs” that can be targeted for development and administration of biological countermeasures.

### Recommendation 4: Integrate the analysis of the immune system with other organs and tissues to preserve the health of the next generation of astronauts

To plan long-lasting deep-space missions, the effects on the immune system and its links with the skin, bone, lungs, microbiome, and cancer following altered gravity and space radiation exposure should be further explored in both humans and animal models. This will require an integrated view by a thorough analysis of the molecular and cellular processes leading to these dysregulations, the sharing of data between specialists in these tissues, and the development of more interdisciplinary projects. Indeed, long-duration space exploration missions might reduce the organism’s ability to respond to injuries, infections, and predispose astronauts to the onset of chronic diseases such as immunosuppression, cardiovascular and metabolic diseases, gut dysbiosis, cancer, and premature aging by strongly influencing redox and metabolic processes, microbiota, immune function, endotoxin, and pro-inflammatory signal production. Furthermore, it is known that low-grade chronic inflammation involves many of the above mechanisms and is a distinctive feature of major chronic diseases. Better knowledge on this topic is therefore needed to manage a number of diseases both in space and on Earth.

## Terestrial benefits and applications

Understanding stressor-related immune challenges in space is highly relevant to the understanding of the biology of cancer immunology, the balance of inflammation and endogenous mechanisms to control it, and the lack of control (autoimmunity/allergies) in the young and ageing population on Earth. Indeed, the abnormal physiology that manifests in healthy humans during their adaptation to space has all the characteristics of accelerated ageing^95^, an observation confirmed in AOS mice^16^. Furthermore, as stressors are omnipresent in our societies which will have to face important climate changes in a near future, this knowledge could contribute to a better understanding of its role in the pathogenesis and pathophysiology of various diseases. Indeed, the functions of the immune system can be affected in response to environmental/living conditions, and chronic and acute stress conditions can result in a further parallel interaction between the immune system and other organ systems. As an example, stressors cause neurophysiologic responses and hormone liberation which can modulate inflammation but also promote bone resorption.

Finally, while the development of personalized medicine, remote controlled flow cytometry and microscopes, biosensors that can measure drug concentration or other biomarkers in minute volumes of blood (or other biological fluids) as well as artificial intelligence/machine learning would be favorable for medical analyses in space, it can also prove valuable at the patient bed site, especially in a medical desert.

## Outlook and summary

A key knowledge gap identified in the process of writing the ESA white paper is the lack of integrating the immune system with other systems such as the musculoskeletal, pulmonary, brain, gut, and cardiovascular systems as well as the close proximity of the immune system with nutrition, metabolism, radiation, and microbes. Therefore, a closer collaboration between researchers involved in space physiology would be desirable to fully understand the physiology of crew members during deep spaceflight. Serious illness of astronauts is so far rare but need to be carefully monitored in preparation for deep-space exploration missions. A recent initiative has suggested a specific immunologic countermeasure protocol for deep-space exploration missions^96^. Importantly, this initiative was focused on precision countermeasures considering the accumulated recent data by integrative analysis of data from different space analogs and samples from ISS crew members. For monitoring the immune system function, cytokines measured in a drop of blood and reactivation of viruses can be measured already. For more complex monitoring of the immune system, technical development of methods is an immediate need. For deep-space exploration, a pre-mission personalized countermeasure protocol should be developed for each crew member and exposure to ground-based facilities will serve an important role in development of such regimen.
